# Truth, Probability, and Frameworks

**DOI:** 10.1371/journal.pmed.0020361

**Published:** 2005-11-29

**Authors:** Jonathan D Wren

**Affiliations:** **1**University of OklahomaNorman, OklahomaUnited States of America

**James T. Kirk:** Harry lied to you, Norman. Everything Harry says is a lie. Remember that, Norman: Everything he says is a lie.

**Harry Mudd:** Now I want you to listen to me very carefully, Norman: I… am… lying.

—*Star Trek*, the episode “I, Mudd”

Although John P. A. Ioannidis [1] brings up several good points about over-reliance on formal—yet arbitrary—statistical cutoffs and bias against the reporting of negative results, his claim that most published research findings are false is somewhat paradoxical. Ironically, the truer his premise is, the less likely his conclusions are. He, after all, relies heavily on other studies to support his premise, so if most (i.e., greater than 50%) of his cited studies are themselves false (including the eight of 37 that pertain to his own work), then his argument is automatically on shaky ground. As mentioned in the *PLoS Medicine* Editorial [2], scientific studies don't offer truth, per se. Even when studies appear in the best journals, they offer probabilistic assertions. Ioannidis's statement that “the probability that a research finding is indeed true depends on the prior probability of it being true” [1] is really begging the question; this, after all, is the problem. We cannot know such probabilities a priori, and guessing at such probabilities and/or parameters (as he does in his single nucleotide polymorphism [SNP] association example) surely could not be less biased than any statistical test of significance. The key problem in Ioannidis's positive predictive value (PPV) formula to calculate the post-study probability that a relationship is true (PPV = [1 − β]*R*/[*R* − β*R* + α], where *R* is the ratio of true relationships to no relationships) is that one can postulate a near-infinite number of non-relationships. Just extending his SNP example, why assume each SNP acts independently? This is not unreasonable, given that schizophrenia is clearly not inherited in a Mendelian pattern. So rather than 99,990 SNPs not being associated with schizophrenia, we have potentially 99,990^*n*^ not associated, where *n* is the number of potentially interacting SNPs. As *n* grows, *R* becomes very small very quickly, and PPV becomes effectively zero. Taken to the extreme, this would imply that all empirical studies are fruitless. One of the most important factors in moving toward the truth, which was not discussed, is fitting discoveries into a framework. Optimally, if a relationship is true, it should have more than one implication, permitting validation from multiple angles. For example, an SNP causally associated with schizophrenia must affect something on the molecular level, whether genomic, transcriptional, post-transcriptional, translational, or post-translational. In turn, these molecules should interact differently with each other, with other molecules within the cell, within a tissue, and/or with the system as a whole. If Norman, the android from *Star Trek* mentioned in the beginning quote, had been equipped with the capacity to evaluate statements within a framework, he never would have short-circuited as a result of Kirk's paradox. He could have entertained the possibility that either Kirk was lying about Harry or Harry's statement was incomplete (i.e., lying about what?) Similarly, repeatedly re-examining any particular finding to resolve the true/not true paradox via statistical arguments alone can short-circuit our patience. We should instead seek to identify the framework by which implications of the finding can be tested, and I would argue that the more important the finding, the more testable implications it has.
